# Using machine learning algorithm to analyse the hypothyroidism complications caused by radiotherapy in patients with head and neck cancer

**DOI:** 10.1038/s41598-023-46509-x

**Published:** 2023-11-06

**Authors:** Tsair-Fwu Lee, Shen-Hao Lee, Chin-Dar Tseng, Chih-Hsueh Lin, Chi-Min Chiu, Guang-Zhi Lin, Jack Yang, Liyun Chang, Yu-Hao Chiu, Chun-Ting Su, Shyh-An Yeh

**Affiliations:** 1 Medical Physics and Informatics Laboratory of Electronic Engineering, National Kaohsiung University of Science and Technology, Kaohsiung, 80778 Taiwan; 2 Department of Electronic Engineering, National Kaohsiung University of Science and Technology, Kaohsiung, 80778 Taiwan; 3 Department of Medical Imaging and Radiological Sciences, Kaohsiung Medical University, Kaohsiung, 80708 Taiwan; 4 PhD Program in Biomedical Engineering, Kaohsiung Medical University, Kaohsiung, 80708 Taiwan; 5 School of Dentistry, College of Dental Medicine, Kaohsiung Medical University, Kaohsiung, 80708 Taiwan; 6 Department of Tactical Control Air Traffic Control & Meteorology, Air Force Institute of Technology, Kaohsiung, 82047 Taiwan; 7 Department of Radiation Oncology, RWJ Medical School, Long Branch, NJ USA; 8 Department of Radiation Oncology, Monmouth Medical Center, RWJBH Medical School, Long Branch, NJ USA; 9 Department of Medical Imaging and Radiological Sciences, I-Shou University, Kaohsiung, 82445 Taiwan; 10 Department of Radiation Oncology, E-DA Hospital, Kaohsiung, 82445 Taiwan

**Keywords:** Cancer, Head and neck cancer

## Abstract

Machine learning algorithms were used to analyze the odds and predictors of complications of thyroid damage after radiation therapy in patients with head and neck cancer. This study used decision tree (DT), random forest (RF), and support vector machine (SVM) algorithms to evaluate predictors for the data of 137 head and neck cancer patients. Candidate factors included gender, age, thyroid volume, minimum dose, average dose, maximum dose, number of treatments, and relative volume of the organ receiving X dose (X: 10, 20, 30, 40, 50, 60 Gy). The algorithm was optimized according to these factors and tenfold cross-validation to analyze the state of thyroid damage and select the predictors of thyroid dysfunction. The importance of the predictors identified by the three machine learning algorithms was ranked: the top five predictors were age, thyroid volume, average dose, V50 and V60. Of these, age and volume were negatively correlated with thyroid damage, indicating that the greater the age and thyroid volume, the lower the risk of thyroid damage; the average dose, V50 and V60 were positively correlated with thyroid damage, indicating that the larger the average dose, V50 and V60, the higher the risk of thyroid damage. The RF algorithm was most accurate in predicting the probability of thyroid damage among the three algorithms optimized using the above factors. The Area under the receiver operating characteristic curve (AUC) was 0.827 and the accuracy (ACC) was 0.824. This study found that five predictors (age, thyroid volume, mean dose, V50 and V60) are important factors affecting the chance that patients with head and neck cancer who received radiation therapy will develop hypothyroidism. Using these factors as the prediction basis of the algorithm and using RF to predict the occurrence of hypothyroidism had the highest ACC, which was 82.4%. This algorithm is quite helpful in predicting the probability of radiotherapy complications. It also provides references for assisting medical decision-making in the future.

## Introduction

Radiation therapy is important in the treatment of head and neck cancer. Because of the use of advanced treatment techniques, there have been considerable improvements in local control and survival rates. However, for surviving head and neck cancer patients, the damage to important normal organs caused by radiation therapy and various symptoms associated with organ damage have a significant impact on normal life functions and quality of life^1^.

Radiation therapy for head and neck cancer patients may damage organs including the brain stem, spinal cord, retina, optic nerve, lens, optic nerve bifurcation, middle ear, inner ear, salivary glands, thyroid, vocal cords, esophagus and other important organs^2^. Clinically, we have observed that, due to thyroid damage after receiving radiation therapy, some patients with head and neck cancer cannot secrete sufficient amounts of thyroid hormones, resulting in hypothyroidism. This is an endocrine disorder, usually associated with abnormal weight gain, high serum total cholesterol concentration, high serum low density lipoprotein cholesterol concentration, tiredness and lethargy, constipation, hair loss, dry skin, chills, irregular menstruation, depression, hoarse voice, memory loss, stunted growth, swelling, slow heartbeat and other symptoms. It has a great impact on the quality of life of patients after surgery^3^.

In the past 20 years, various artificial intelligence techniques and feature selection algorithms have been used in the predictive analysis of cancer prognosis^4^. A high proportion of these studies used machine learning to construct cancer analysis algorithms, and supervised learning has been used in classification algorithms for feature labeling^5^.

Ma et al. highlighted 5-year survival and late toxicity data from a nonprofit, multi-institutional, prospective, open-label, randomized phase 2 trial on survival associations with radiation therapy in patients with head and neck cancer. The study helps explain the impact of radiation therapy on patients with head and neck cancer^1^. Mario explained the advancement of deep learning technology in cancer treatment in new directions of artificial intelligence in cancer imaging^4^. In addition, Huang, S. et al. researched on the application of artificial intelligence in cancer diagnosis and prognosis emphasizes that artificial intelligence is applied to assist cancer diagnosis and prognosis. The accuracy (ACC) is higher than that of general statistical experts^5^.

**Figure 1:**
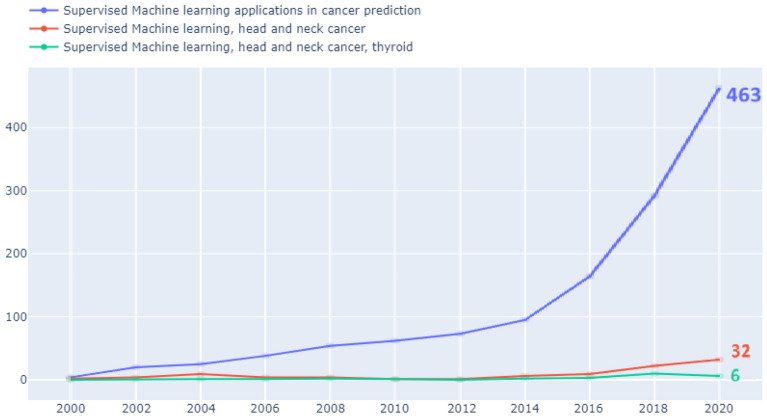
Trends in almost 20 years of research articles using supervised techniques for cancer predictive analysis, head and neck cancer research and thyroid research based on keyword combinations.

Figure 1 depicts trends in the number of published journal articles using supervised learning techniques in (a) prediction in cancer analysis, (b) head and neck cancer, and (c) thyroid studies in head and neck cancer patients. These publications were identified based on a combination of various keywords in Science Direct’s online database of global scientific research publications^6^. In addition to excluding articles published before 2000, the search was limited to journal research articles. As mentioned above, it can be seen from Fig. 1 that the application of supervised learning algorithms to thyroid research in patients with head and neck cancer is obviously insufficient, with fewer than 10 journal papers published annually worldwide from 2000 to 2020^7^.

The Lyman Kutcher-Burman (LKB)^8^ algorithm is often used clinically to assess the normal tissue complication probability (NTCP). However, the standard deviation of the data samples in this study was too large to use the NTCP algorithm in an optimal fashion^9^. Supervised machine learning was chosen for use instead. Considering that the analysis of the dose factor alone cannot take into account the comprehensiveness, only a single dose can be used for each analysis. Most studies performed dose prediction analysis only for the 50% toxic dose (TD50). Factors other than dose were not explored. In view of the above, the current study adopted machine learning (ML) algorithms in artificial intelligence (AI) as the analysis technology^10^.

There are two main reasons for choosing machine learning algorithms such as decision trees (DT), random forest (RF), and support vector machines (SVM) in this study. One is that the tree model-based algorithm is easier to interpret than other algorithms, and the prediction results are easier to understand. The second is that SVM achieve accurate prediction performance under the nonlinear relationship between features and results, and are popular in machine learning and related applications. A geometric framework is suitable for classification problems and provides an intuitive basis for a clear understanding of geometric optimization algorithms. Thus, a practical solution is provided for practical classification projects.

Since existing clinical medical research rarely mentions the predictors of thyroid damage caused by intensity-modulated radiation therapy for head and neck cancer, the purpose of this study was to identify such predictors.

## Materials and methods

One hundred and forty patients with head and neck cancer were treated with radiotherapy at a medical center, Department of Radiation Oncology, E-DA Hospital, Kaohsiung, Taiwan. The data was anonymized prior to the authors obtaining it. The rationale behind the chosen study period was mainly our manpower. A longer chosen study period means more patient data will be included. We started this analysis since 2021. Our prior study showed the median value of the interval between the completion of radiotherapy and the first occurrence of biochemical hypothyroidism was 29 months. Therefore, we chose the interval of March 2015 to March 2016 to ensure that at least five years of follow-up has elapsed.

The software and numbers used for statistics and analysis are as follows: Python 3.7.4 [MSC v.1915 64 bit (AMD64)], RStudio Version 1.2.5033.

Intensity Modulated Radiation Therapy (IMRT) treatment planning by Varian Eclipse software (Varian Medical Systems, Palo Alto, California, USA) was applied for all patients. The 140 patients in the database had no distant metastases and all completed the course of radiation therapy. Three patients with extreme values of radiation dose volume data were excluded. Biochemical hypothyroidism was defined as thyroid-stimulating hormone (thyrotropin) value > 5.0 mIU/L and/or free tetraiodothyronine < 0.7 ng/dL. There were 105 patients (76.6%) with biochemical hypothyroidism and the interval between the completion of radiotherapy and the first occurrence of biochemical hypothyroidism ranged from 7.2 to 70 months (median: 29.0 months). None of the patients had hypothyroidism or any other thyroid or thyroid defect before radiation therapy. Institutional Review Board (IRB) approval was obtained from the E-DA Hospital IRB (approval number: EMRP-103-063). The IRB waived informed consent requirement of the study because it was a retrospective work. We confirm that all methods were performed in accordance with relevant regulations and test guidelines.

Table 1 presents the clinical factors and related information for the 137 patients included. These data were used to assess whether patients developed hypothyroidism as a consequence of therapy, defined as high TSH = 1, a thyrotropin blood concentration greater than 5.0 mIU/L or a free tetraiodothyronine blood concentration of less than 0.7 ng/dL.

**Table 1:** Patient’s clinical factors.

Factors	Value-x (%) HNC (n = 137)
Age (years)	
Mean	56
Range	37–81
Gender (n)	
Male	129 (94.2%)
Female	8 (5.8%)
Fraction (time)	
Mean	32
Range	30–35
Tumor site	
Larynx	1 (0.7%)
Hypopharynx	20 (14.6%)
Oropharynx	37 (27.0%)
Oral cavity	79 (57.7%)
T category	
T1	17 (12.4%)
T2	36 (26.3%)
T3	27 (19.7%)
T4a	50 (36.5%)
N category	
N0	49 (35.8%)
N1	16 (11.7%)
N2	70 (51.1%)
N3	2 (1.4%)
AJCC stage	
I	1 (0.7%)
II	9 (6.6%)
III	18 (13.1%)
IV	109 (79.6%)
Hypothyroidism	
Yes	105 (76.6%)
No	32 (23.4%)

*AJCC* American Joint Committee on Cancer, *HNC* head and neck cancers.

Risk factors affecting hypothyroidism after radiotherapy can be divided into dose factors and clinical factors. Dose factors for patients are shown in Table 2. Clinical factors include age, gender and number of treatments (fraction), as mentioned in Table 1. The above risk factors were substituted into three algorithms for analysis and prediction.

**Table 2:** Patient dose factors.

Factors	highTSH = 0 (n = 105)	highTSH = 1 (n = 32)
Dose (Gy)		
Mean (range)	63.44 (54–70)	64.25 (60–70)
Volume (mL)		
Mean (range)	15.41 (4.3–48.8)	9.89 (1.8–22.4)
DMean (Gy)		
Mean (range)	55.04 (0.67–67.15)	61.24 (54.85–71.51)
V10 (%)		
Mean (range)	95.62 (0–100)	100 (100–100)
V20 (%)		
Mean (range)	95.36 (0–100)	100 (100–100)
V30 (%)		
Mean (range)	94.20 (0–100)	100 (99.97–100)
V40 (%)		
Mean (range)	93.58 (0–100)	99.99 (90.54–100)
V50 (%)		
Mean (range)	88.61 (0–100)	98.87 (90.54–100)
V60 (%)		
Mean (range)	31.76 (0–99.99)	54.72 (0–100)
DMax (Gy)		
Mean (range)	65.8 (3.03–77.28)	68.51 (59.92–76.97)

*highTSH* high thyrotropin, *Vx* relative volume of organs receiving X doses, X = 10, 20, 30, 40, 50, 60.

**Figure 2:**
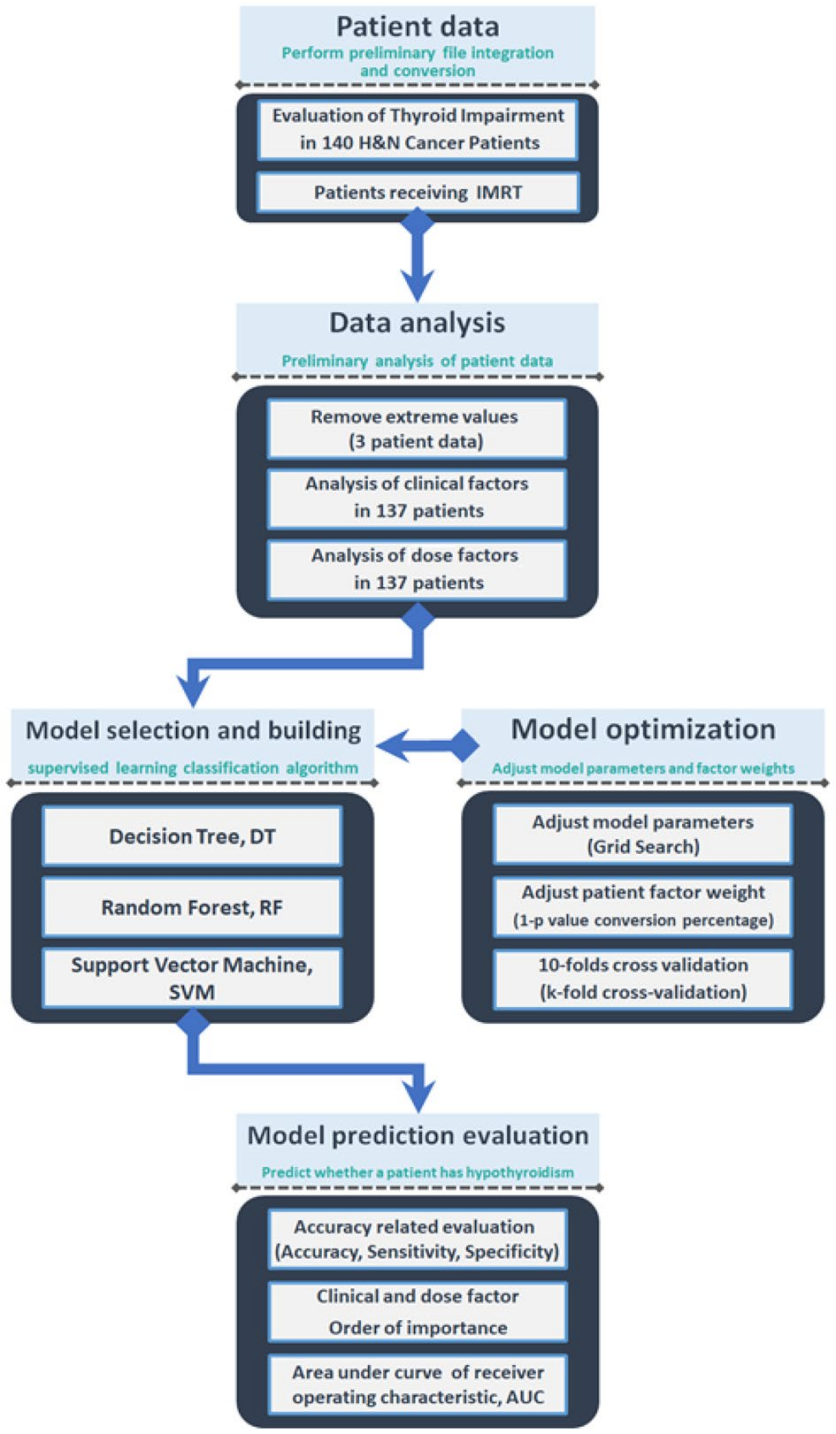
Research general flow chart.

Figure 2 shows the research flow of this study. Patient data were classified one by one for preliminary data sorting. Models suitable for data analysis by data type were evaluated, including DT, RF and SVM. Patient data were substituted into the model to select important factors and predict hypothyroidism. Individual algorithms were optimized. Methods included Grid Search and K-fold cross-validation. Finally, the results show the importance ranking and prediction ACC of the factors selected by the algorithms, which were evaluated by statistical indicators common to machine learning algorithms.

Among machine learning algorithms, tree model-based algorithms have excellent predictive performance for two main reasons: (a) The interpretability of the results is high^11–14^. That is, it is possible to understand the contribution of each feature to the predicted outcome; (b) by enhancing overall learning^15^, it is possible to improve the predicted outcome of the decision tree-based classification^16^. Therefore, this study used two widely used algorithms based on tree models: DT and RF. The third algorithm used was the SVM algorithm^17^. This is popular in machine learning and related applications due to its ability to achieve accurate prediction performance under the nonlinear relationship between features and results. It uses mathematical transformations of kernel tricks, and thus applies to the geometric framework of classification problems and provides an intuitive basis for a clear understanding of geometric optimization algorithms. Thus, a practical solution is provided for practical classification projects^18^.

Binary logistic regression analysis was used to confirm the significance of correlations between the input eigenfactors and the output factors. In order to confirm the positive and negative correlation between input and output, the Pearson product-difference correlation coefficient was used as a reference for focusing on the correlation between input factors with a significant influence and thyroid damage.

The grid search method and the theory of the algorithm were used to adjust the optimization of the parameters of the algorithm. Data feature factor weights were percentage-transformed based on 1-p values^19^. Post-pruning was used in the DT algorithm to adjust the optimized decision tree parameters. RF was optimized by analyzing the error rate and mtry parameter adjustment through a preliminary algorithm. The SVM part adjusted the cost and gamma values in the parameters of the algorithm, and arranged and combined the parameters for optimization. Ten crosses were used to verify the fit of the algorithm using relevant indicators such as validation ACC^20^.

Grid search is a method of optimizing the performance of an algorithm by traversing a given combination of arguments. An exhaustive search method for specifying parameter values. At the same time, in order to avoid the overfitting of the algorithm, the tenfold cross-validation method was adopted in this study: the patient dataset was randomly divided into 10 groups for cross-validation. The data from nine groups was used as the training set, and the remaining group was used as the validation set. Repeat the substitution into the module for 10 verifications. The ratio of training set, validation set, and test is based on the common standard of 6:2:2. The ranking results of factor importance are shown in Fig. 2.

### Institutional review board statement

Institutional Review Board (IRB) approval was obtained from E-Da Hospital (Approval Number: EMRP-103-063). The IRB waived informed consent requirement of the study because it was a retrospective work.

### Informed consent

All authors have confirmed the manuscript and approved the publication of the manuscript.

## Results

The ranking of important factors selected by each algorithm and the statistical indicators of each algorithm are shown to illustrate the ACC of the prediction. The relationship between the factors is shown by the Pearson coefficient.

### Order of importance of factors

**Figure 3:**
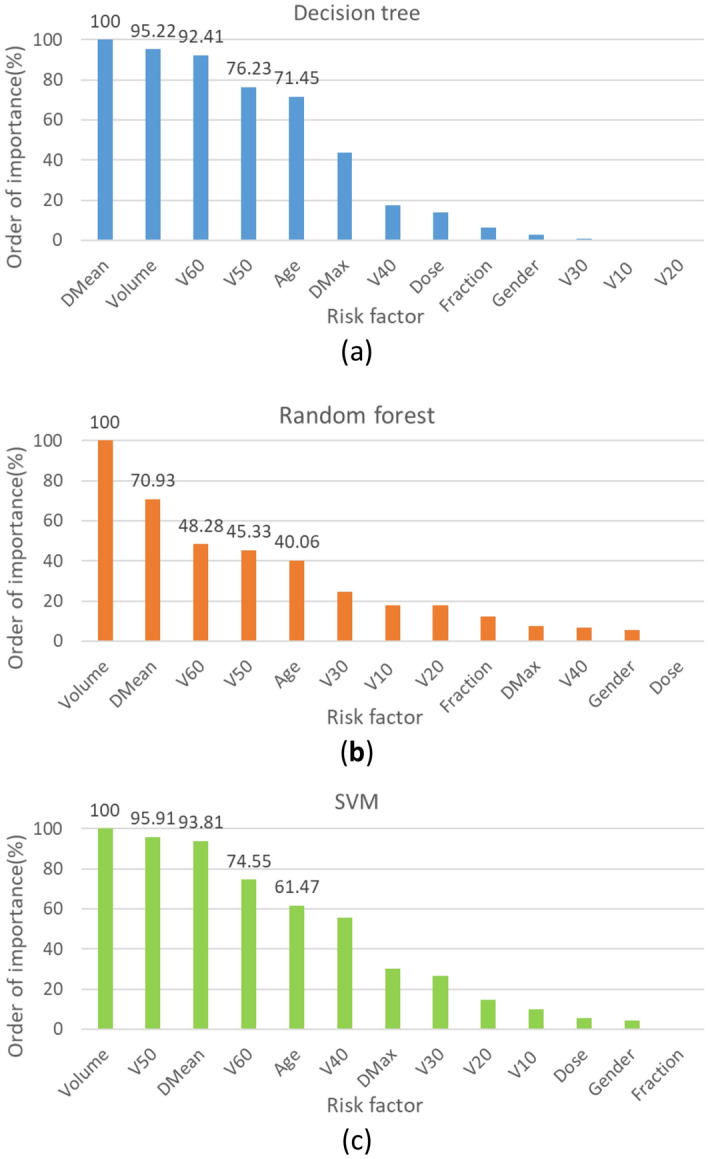
Feature importance ranking percentage of three algorithms (**a**) decision tree, (**b**) random forest, (**c**) support vector machine.

According to the importance ranking of clinical and dose factors selected by the three algorithms (Fig. 3), we found that although the factors selected by DT, RF and SVM were ranked differently, the same top five factors were Volume, Dmean, Age, V50 and V60 in each algorithm, suggesting that they have the greatest impact on hypothyroidism after radiotherapy.

### Algorithm evaluation

Based on the analysis of statistical indicators and the importance ranking of each algorithm, after adjusting the weights and parameters, the overall algorithm ACC, training set ACC, test set ACC and related statistical indicators of the three optimized parameters were compared and sorted, as shown in Table 3. The model metrics in Table 3 show the predictive power of the three algorithms. Among them, RF showed the best stability with an AUC of 0.827.

**Table 3:** Statistical data comparison of three algorithms.

Model	ACC (95 CI)	Sensitivity	Specificity	Balanced Accuracy	Prevalence	AUC
Decision Tree	0.834 (0.5564, 0.8712)	0.840	0.444	0.295	0.735	0.794
Random Forest	0.912 (0.6547, 0.9324)	0.833	0.750	0.407	0.882	0.827
SVM	0.800 (0.621, 0.913)	0.828	0.600	0.343	0.853	0.692

*AUC* area under curve, *CI* confidence interval, *SVM* support vector machine.

### Relationship between factors

**Figure 4:**
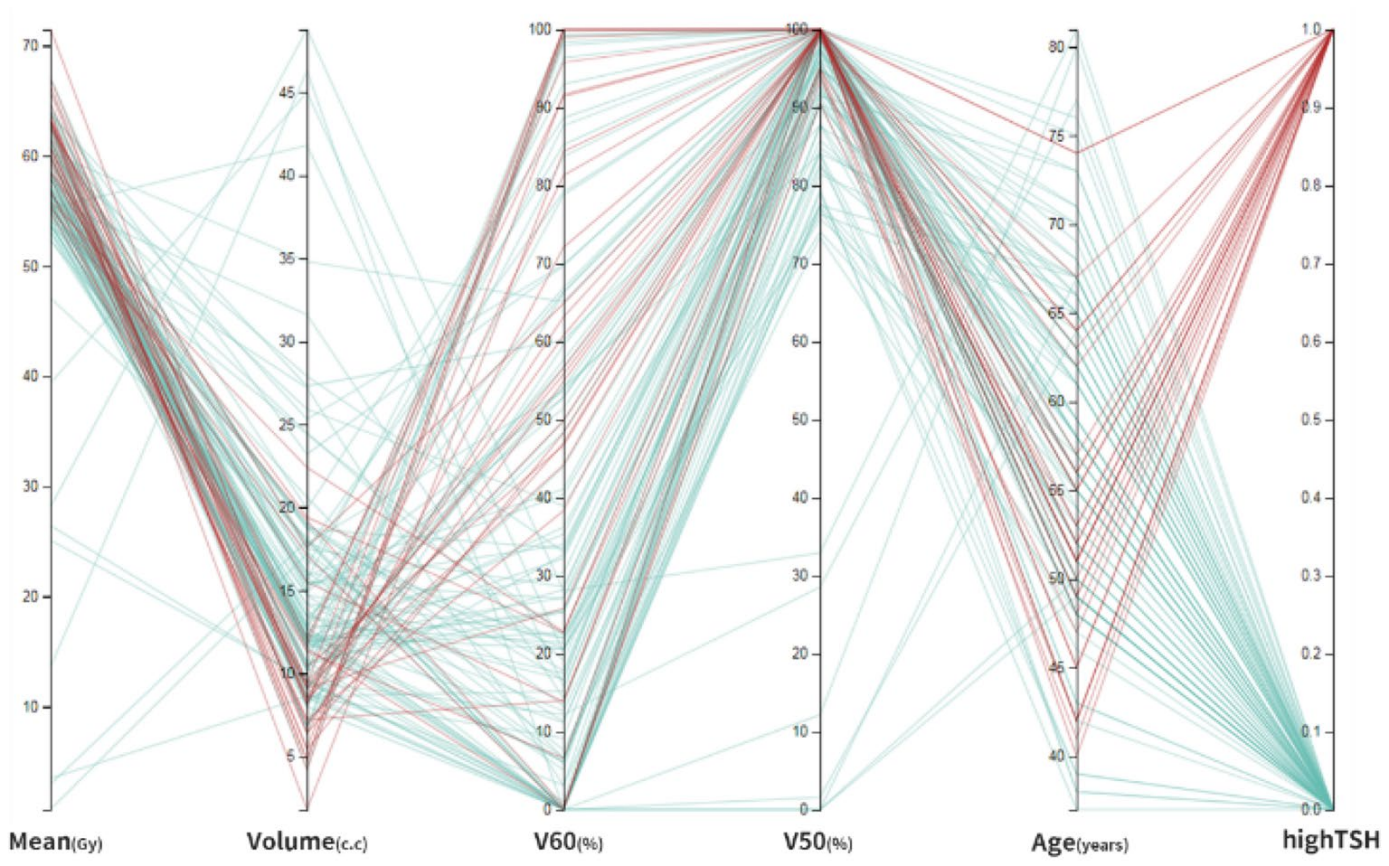
Significant factors in patient data.

Figure 4 is a parallel plot of significant factors in patient data. Much can be learned about the data. In terms of the relationship between the average dose, volume factor, V50, V60 and age, it can be seen that the smaller the volume, the more likely the patient is to have damage to the thyroid in the later line segment. The higher the dose factors of V50 and V60, the easier it is to cause thyroid damage. In the parallel coordinates of age and thyroid damage, it can be seen that the younger the age, the higher the tendency for thyroid damage. The Pearson product-difference correlation coefficient indicated the correlation between age, volume factor, mean dose, V50, V60 dose factor and output. Age was negatively correlated with the volume factor, and the mean dose, V50 and V60 were positively correlated.

## Discussion

In this study, we used the above three machine learning models to analyze the complication rates of thyroid damage after radiation therapy in patients with head and neck cancer. After parameter optimization and tenfold cross-validation, the best prediction ability was achieved by RF, with an AUC of 0.827. In a previous study Jamie et al. used penalised logistic regression (PLR), support vector classification (SVC) and random forest classification (RFC) algorithms to predict the model of mucositis caused by head and neck radiotherapy, which was cross-validated for 100 iterations^21^. Their results showed that the RFC standard, which does not contain spatial information, had the best ACC. As far as the data type is concerned, they used radiation dose as a feature to substitute into the model for prediction. This is the same type of continuous variable as the dose factor used in this study, which may indicate that RF can achieve a good prediction effect using similar data types.

Dean et al. used SVC, RFC and logistic regression classification (LRC) to build a predictive model for dysphagia induced by radiation therapy for head and neck cancer^22^. Organs at risk (OAR) dose parameters were used as features for prediction. The results showed that the AUC of RFC was still the highest among the three algorithms. From the above results, it can be seen that the performance of the random forest algorithm as a prediction model for complications can be considered excellent.

The analysis of risk predictors in this study was performed by ranking their importance using three machine learning algorithms. All three algorithms selected the same five predictors: age, thyroid volume, mean dose, V50 and V60. However, the ranking of the above five factors differed for the three algorithms, due to the different decision functions and parameters used by the algorithms.

Age was negatively correlated with the size factor, indicating that the greater the age and thyroid volume, the lower the risk of thyroid damage. The mean dose, V50, and V60 were positively correlated, indicating that the greater the mean dose, V50, and V60, the higher the risk of thyroid damage. By analyzing the results of machine learning and evaluating the statistical indicators, age, thyroid volume, mean dose, V60, and V50 were found to be related to the occurrence of hypothyroidism.

We found that older patients were less likely to develop hypothyroidism. This contrasts with many other radiation therapy-related side effects associated with age. Generally, older patients are less likely to experience side effects associated with radiation therapy than younger patients due to the aging of cells and tissues. Other studies have reported similar findings^23^. The exact turnaround is still unclear, and more research is needed in this respect.

Thyroid volume is also an important factor in the development of hypothyroidism after radiation therapy. This study found that patients with a larger thyroid volume before radiation therapy were less likely to develop hypothyroidism. This may be because the larger the volume of the thyroid, the lower the percentage of the entire thyroid that is covered by high doses of radiation. This shows that more thyroid glands receive only low doses of radiation and their function is less susceptible to damage. Other studies have reported similar findings^24^.

It has been reported in the literature that radiation dose is associated with the occurrence of hypothyroidism^25^. The computerized treatment planning system in radiation therapy will calculate the highest radiation dose, the lowest radiation dose and the average radiation dose received by the thyroid gland. This study found an association between the average radiation dose received by the thyroid and the development of hypothyroidism after radiation therapy. The higher the average radiation dose received by the thyroid, the higher the chance of developing hypothyroidism after radiation therapy. Other studies have reported similar findings^26^.

When discussing side effects associated with radiation therapy, both the radiation dose and the volume of tissue receiving the radiation dose influence the outcome. This study found that two factors of radiation dose and volume irradiated were associated with the development of hypothyroidism after radiation therapy. These two factors were the volume of the thyroid gland receiving a radiation dose of 60 Gy or more (V60 Gy), and the volume of the thyroid gland receiving a radiation dose of 50 Gy or more (V50 Gy). This study found that the larger the volume of the thyroid that received radiation doses greater than or equal to 60 Gy (V60 Gy), the higher the probability of developing hypothyroidism after radiation therapy. The larger the volume (V50 Gy) of the thyroid that received a radiation dose of 50 Gy or more, the higher the probability of developing hypothyroidism after radiation therapy. This means that both volume and radiation dose play an important role, perhaps additively, in causing damage to thyroid tissue. Other studies also have reported similar findings^27^.

Because hypothyroidism has a great impact on the health and quality of life of patients, the question of how to prevent hypothyroidism caused by radiation therapy is an important topic. Previous studies gave patients high doses of thyroid hormone during radiation therapy, and found that the patients’ thyroid-stimulating hormone and thyrotropin-releasing hormone decreased. This reduction allows the thyroid cells to be in a metabolically quiescent condition, and makes them less susceptible to radiation damage^28^. These studies found a reduction in the incidence of hypothyroidism, but similar studies found that giving patients high doses of thyroid hormone did not reduce the incidence of hypothyroidism^29^.

Other researchers have questioned whether irradiation to the thyroid gland can be avoided entirely. However, because the thyroid gland is next to the third and fourth regions of the neck lymph nodes, these lymph nodes are included in the target field when carrying out radiation therapy for head and neck cancer. To reduce the occurrence of hypothyroidism, the radiation dose of the irradiation target area would have to be lowered, and the conformal coating would not be as good, which could lead to tumor recurrence^30^.

Therefore, it is currently suggested that the ideal approach is to give appropriate dose volume limitation to the thyroid when performing computerized radiation therapy planning optimization. The occurrence of hypothyroidism can be reduced as much as possible without affecting the radiation dose conformal coating of the irradiated target area. The mean dose of radiation received by the thyroid gland found in this study, the volume of the thyroid receiving a radiation dose greater than or equal to 60 Gray (V60), and the volume of thyroid receiving a radiation dose greater than or equal to 50 Gray (V50), could provide a reference for establishing a consensus on the radiation dose/volume constraint of the thyroid in the future.

Research limitations posed by skewed class distributions. In future research, appropriate algorithms or techniques should be used to deal with unbalanced data sets. Additionally, the lack of data in this study is another limitation. This may include its limitations on the scope of our analysis, potential sampling bias, or making the conclusions less statistically powerful. The potential impact of these limitations on the reliability and validity of our results.

There are several limitations of our research. There was at least five years of follow-up for the 140 patients. Our prior study of patients with nasopharyngeal cancer treated with IMRT techniques showed the interval between the completion of radiotherapy and the first occurrence of biochemical hypothyroidism ranged from 6.1 to 99.4 months (median: 29.0 months). A longer follow-up period might reveal more patients with hypothyroidism. However, considering the manpower and data storage facility, we chose the cohort of patients treated between 2015 and 2016. Another shortcoming is the retrospective nature of this research. We could not avoid the inherent disadvantages of retrospective study. For retrospective study of machine learning, the training and validation are performed on the existing datasets. However, for a prospective research, as with retrospective study, the training is performed on an existing database, but the validation process is performed on newly collected data. In general, a prospective research will be more likely to correctly validate the real-world performance of machine learning models. In the future, based on current trained model, we would like to design a research to prospectively and externally validate the machine learning model.

Past studies include Ren et al. on radiation-induced hypothyroidism in patients with nasopharyngeal carcinoma^31^, Kim et al. on dose-volume parameters for predicting hypothyroidism after radiotherapy for head and neck cancer^32^, and Zhou et al. on radiation-induced hypothyroidism in head and neck cancer^33^. These studies are similar to this study in that they are all studies of diseases caused by radiation therapy. However, the algorithm used is not exactly the same as in this study. The types of diseases studied were also different. Therefore, there are differences in the results of the studies.

Until now, there has been no clear evidence that cisplatin and fluorouracil have significant impact on the occurrence of thyroid disorders. Mercado et al. ever retrospectively analyzed the data from a randomized trial and concluded that administration of cisplatin and fluorouracil concurrently with radiotherapy did not increase the risk of hypothyroidism relative to radiotherapy alone^34^. Sinard et al. designed a prospective study to assess the incidence and time frame of occurrence of hypothyroidism in patients by primary tumor site and treatment modality^35^, and demonstrated that the difference of incidence of hypothyroidism in patients with and without chemotherapy was not statistically significant.

In our country, male accounts for more than 90% of the head and neck cancer patients. Therefore, our results might not be generalizable to be applied to female patients with head and neck cancers. However, our data are useful for the majority of head and neck cancer patients.

The machine learning models were selected for this study due to their interpretability. However, clinicians' understanding of machine learning models needs improvement through various methods. These methods aim to provide interpretable and actionable insights for healthcare professionals who may not be familiar with advanced computing technologies. These include simplified technical terminology, visual explanations, clinical scenarios, and user-friendly interfaces.

## Conclusions

Clinically, we have observed that some patients with head and neck cancers undergo radiation therapy, which result in thyroid damage and hypofunction. In past studies, various artificial intelligence techniques and feature selection algorithms were used for predictive analysis of cancer prognosis. In this study, DT, RF and SVM were selected as the main research algorithms, and all identified age, thyroid volume, Dmean, V60, and v50 as important predictors of thyroid damage. For each of the three algorithms in this study, the grid search method was used to optimize the algorithm to improve its ACC and other reference indicators such as AUC. Among the three algorithms, RF performed the best, achieving an AUC and ACC above 0.8. Research on thyroid damage in head and neck cancer has been relatively rare in recent years, but the five factors that affect the thyroid and the occurrence of hypothyroidism found in this study provide a reference for research related to the treatment of head and neck cancer. It is also hoped that in the future, they could be used to evaluate the data of patients receiving radiotherapy, and at the same time, provide reference indicators for clinicians in treatment planning.

## Data Availability

The datasets used and analysed during the current study available from the corresponding author on reasonable request. Because of legal restrictions and ethics, the data in this manuscript are available upon formal request from the corresponding author.
